# HTLV-1 drives vigorous clonal expansion of infected CD8^+^ T cells in natural infection

**DOI:** 10.1186/s12977-015-0221-1

**Published:** 2015-11-09

**Authors:** Anat Melamed, Daniel J. Laydon, Hebah Al Khatib, Aileen G. Rowan, Graham P. Taylor, Charles R. M. Bangham

**Affiliations:** Section of Virology, Imperial College London, Wright-Fleming Institute, Norfolk Place, London, W2 1PG UK

**Keywords:** Human retroviral infection, HTLV-1, Clonality, Integration, Cytotoxic T cells, Latency

## Abstract

**Background:**

Human T-lymphotropic Virus Type I (HTLV-1) is a retrovirus that persistently infects 5–10 million individuals worldwide and causes disabling or fatal inflammatory and malignant diseases. The majority of the HTLV-1 proviral load is found in CD4^+^ T cells, and the phenotype of adult T cell leukemia (ATL) is typically CD4^+^. HTLV-1 also infects CD8^+^ cells in vivo, but the relative abundance and clonal composition of the two infected subpopulations have not been studied. We used a high-throughput DNA sequencing protocol to map and quantify HTLV-1 proviral integration sites in separated populations of CD4^+^ cells, CD8^+^ cells and unsorted peripheral blood mononuclear cells from 12 HTLV-1-infected individuals.

**Results:**

We show that the infected CD8^+^ cells constitute a median of 5 % of the HTLV-1 proviral load. However, HTLV-1-infected CD8^+^ clones undergo much greater oligoclonal proliferation than the infected CD4^+^ clones in infected individuals, regardless of disease manifestation. The CD8^+^ clones are over-represented among the most abundant clones in the blood and are redetected even after several years.

**Conclusions:**

We conclude that although they make up only 5 % of the proviral load, the HTLV-1-infected CD8^+^ T-cells make a major impact on the clonal composition of HTLV-1-infected cells in the blood. The greater degree of oligoclonal expansion observed in the infected CD8^+^ T cells, contrasts with the CD4^+^ phenotype of ATL; cases of CD8^+^ adult T-cell leukaemia/lymphoma are rare. This work is consistent with growing evidence that oligoclonal expansion of HTLV-1-infected cells is not sufficient for malignant transformation.

**Electronic supplementary material:**

The online version of this article (doi:10.1186/s12977-015-0221-1) contains supplementary material, which is available to authorized users.

## Background

The retrovirus Human T-Lymphotropic Virus Type I (HTLV-1) causes a life-long infection in an estimated 5–10 million individuals world-wide, resulting in disabling or fatal inflammatory and malignant diseases in ~10 % of infected people [1]. It is not completely understood what determines an individual’s risk of these HTLV-1-associated diseases; however, a high proviral load (PVL; the number of proviral copies per 100 cells) in peripheral blood mononuclear cells (PBMCs) is correlated with the risk of both the central nervous system inflammatory disease known as HTLV-1-associated myelopathy/tropical spastic paraparesis (HAM/TSP) and the malignant disease adult T-cell leukemia/lymphoma (ATL) [2, 3].

HTLV-1 can infect most nucleated mammalian cells in vitro, including both CD4^+^ and CD8^+^ T cells, but in vivo the virus is predominantly found in CD4^+^ T cells [4, 5]. The reasons for this preferential carriage in CD4^+^ T cells in vivo are not clear; mechanisms related to the cell-type distribution of cellular receptors for HTLV-1 [6] and to long-term selection in vivo [7] have been suggested. ATL is typically a malignancy of CD4^+^ cells [8, 9]. The standard model of HTLV-1-driven cell transformation focuses on life-long clonal expansion of HTLV-1-infected CD4^+^ cells as a precursor to malignancy [10].

HTLV-1-infected CD8^+^ cells may have great importance. Tax-specific CD8^+^ cells are themselves more likely than CD8^+^ cells specific to another virus to be infected with HTLV-1 [11]. Virus-specific CD8^+^ cells can both exert a protective antiviral effect and contribute to the pathogenesis of viral diseases such as HAM/TSP. It is unknown which of the effects attributed to the Tax-specific CD8^+^ cells result from their infection status, and there are conflicting reports in the literature on their functionality [12–14].

High-throughput analysis of proviral integration sites [15] has given new insights into the integration site preferences and frequency distribution of HTLV-1- infected clones in asymptomatic carriers (AC) of the virus and in patients with the different disease manifestations [15–17]; the relationship between integration site, HTLV-1 clonality, proviral expression and the immune response [18, 19]; and the integration site and clonality in related retroviruses [20–22]. Since there is typically a single integrated HTLV-1 provirus per cell [23] the number of HTLV-1-infected clones can be quantified by the abundance of observed integration sites.

Previous analyses of HTLV-1 integration were carried out on populations of unsorted PBMCs, and so did not distinguish between the different cell populations, in particular CD8^+^ and CD4^+^ T cells. The objective of the current study was to analyse the clonality of HTLV-1-infected CD4^+^ and CD8^+^ cells in both unsorted PBMCs and purified CD4^+^ and CD8^+^ populations, and quantify the contribution to the HTLV-1 proviral load made by each respective population.

## Results

### Five percent of the proviral load of HTLV-1 is carried by CD8^+^ cells

In order to separate CD4^+^ and CD8^+^ cells, purified PBMCs from 12 HTLV-1-infected subjects (6 ACs and 6 patients with HAM/TSP; Table 1) were magnetically sorted on the basis of cell surface expression of CD4 or CD8. The purity of sorted samples was measured by flow cytometry, and DNA extracted from the sorted and unsorted populations was used to assay the proviral load by quantitative PCR (qPCR) and to analyse clonality by high-throughput sequencing. We used the clonality analysis to study each sorted cell population separately, and to determine whether clones identified in the unsorted (PBMC) population were CD4^+^ or CD8^+^. We attributed 72.8 % of all clones to either CD4^+^ or CD8^+^ cells (Additional file 1: Figure S1). Clones which were not attributed had a significantly lower absolute abundance and were therefore less likely to be detected in both the sorted cells and the PBMCs. The median frequency of contaminating CD4^+^ cells in the CD8^+^ fraction was 0.47 % (range 0.07–2.07 %) and the median frequency of contaminating CD8^+^ cells in the CD4^+^ fraction was 0.94 % (0.43–5.44 %).

**Table 1:** Subject samples used in this work

Subject		Known comorbidities	Absolute cell counts (cells/µl)		Populations within T cells (%)		Populations in PBMC (%)	
Code	Clinical diagnosis		CD4^+^	CD8^+^	CD4^+^/CD3^+^	CD8^+^/CD3^+^	CD3^+^CD4^+^ in total	CD3^+^CD8^+^ in total
HBX	AC	None	NA	NA	66.3	29.5	30.8	13.7
HBZ	AC	None	948	284	74.1	21.1	41.2	11.7
HCP	AC	None	732	335	60.5	29.4	22.1	10.7
HEZ	AC	Hepatitis^a^	631	214	60.8	29.1	27.9	13.3
HGL	AC^b^	None	835	378	67.5	28.6	28.3	12
HHD	AC	None	1316	419	71.5	24.4	33.4	11.4
TAN	HAM/TSP	None	588	260	60.8	32.5	24.8	13.3
TAZ	HAM/TSP	None	1560	1058	46.4	42.5	29.3	26.8
TBW	HAM/TSP	None	300	1254	16.4	81.1	12.2	60.3
TDB	HAM/TSP	None	838	406	56.2	33.3	22.6	13.4
TDL	HAM/TSP	Shingles	1526^c^	707	60.6	34.8	32	18.3
TDT	HAM/TSP	None	752	392	59.2	32.5	29.7	16.3

PBMC samples from 12 HTLV-1-infected individuals were examined in this work

Details of the CD4^+^, CD8^+^ cell populations are shown here

^a^ *HEZ* Hepatitis of unknown origin (negative for HCV, HBV)

^b^ *HGL* considered to be asymptomatic carrier at time of blood sample, but was diagnosed with HAM/TSP about a year later

^c^ *TDL* absolute cell counts from an earlier timepoint (1 month earlier)

The proviral load in the sorted and unsorted populations (Additional file 2: Table S1) was measured using qPCR. As expected in this cohort, unsorted cells had a high proviral load (median 5 copies, range 3.7 to 11.33 copies per 100 PBMCs). In the samples sorted for CD4^+^ or CD8^+^ cells, the median proviral load was 12.3 copies (6.0–30.2) and 2.0 (1.1–6.2) copies per 100 cells, respectively.

**Fig. 1:**
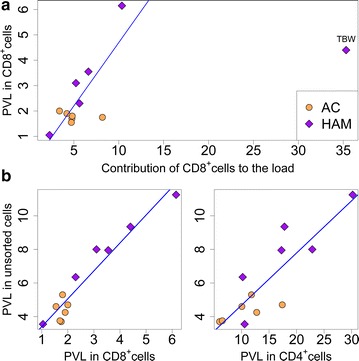
Five percent of HTLV-1 proviral load is carried in CD8^+^ cells. HTLV-1-infected CD4^+^ and CD8^+^ cells were separated by magnetic bead sorting and analysed for their HTLV-1 proviral load and integration site frequency. **a** The number of proviral copies per 100 CD8^+^ cells and the percentage of contribution of CD8^+^ cells to the proviral load was quantified in 12 HTLV-1 carriers. The median percentage of the load carried by CD8^+^ cells was 5 %. A significant positive correlation was found between the proportion of the total HTLV-1 proviral load in PBMCs that was carried by CD8^+^ cells and the proviral load in these cells (p = 0.01, Spearman’s rank correlation). Regression line based on linear regression excluding the CD4^+^ lymphopenic outlier (TBW); see text for details. **b** The proviral load (PVL, copies per 100 cells) in unsorted PBMCs was strongly correlated with the proviral load in both CD8^+^ cells and CD4^+^ cells (p < 0.0001 and p = 0.004, respectively, Spearman’s rank correlation)

The proportion of the load carried by the CD8^+^ cells was calculated from the proviral load measured and the proportion of CD8^+^ cells in each population. The median proportion of the proviral load present in CD8^+^ cells was 5.02 % (range 2.29–35.32 %, Fig. 1a; Additional file 2: Table S1). This estimate was confirmed using the high-throughput sequence data, by using the proportion of all proviruses in the unsorted samples attributed to CD8^+^ clones. There was a strong linear correlation between the estimates from the two independent approaches (Additional file 3: Figure S2, Pearson linear regression, p < 0.0001, r = 0.969). An exceptionally high proportion of the load was carried in CD8^+^ cells in one case of HAM/TSP (subject code TBW). This HIV-seronegative subject has a chronic idiopathic CD4^+^ lymphopenia leading to an extremely low CD4^+^/CD8^+^ ratio in his circulating T cells (Table 1). In this case, approximately 35 % of the proviral load in the blood was carried in CD8^+^ T-cells. Due to the unique nature of the infection in this subject statistical analysis was carried out both including and excluding this case which did not alter our conclusions (Additional file 2: Table S3).

The contribution of CD8^+^ cells to the load was significantly correlated with the proviral load in unsorted cells and with the proviral load in CD8^+^ cells (p = 0.02 and p = 0.01 respectively, Spearman’s rank correlation, Fig. 1a). There was no correlation between the proviral load in CD4^+^ cells and the contribution of CD8^+^ cells to the load.

### HTLV-1-infected CD8^+^ cells are highly oligoclonal

We wished to compare the degree of oligoclonality between the infected CD8^+^ cells and the infected CD4^+^ cells in each subject. The proviral load in PBMCs was strongly correlated with both the proviral load in CD8^+^ cells (p < 0.0001, Spearman’s rank correlation) and the proviral load in CD4^+^ cells (p = 0.004, Spearman’s rank correlation) (Fig. 1b).

**Fig. 2:**
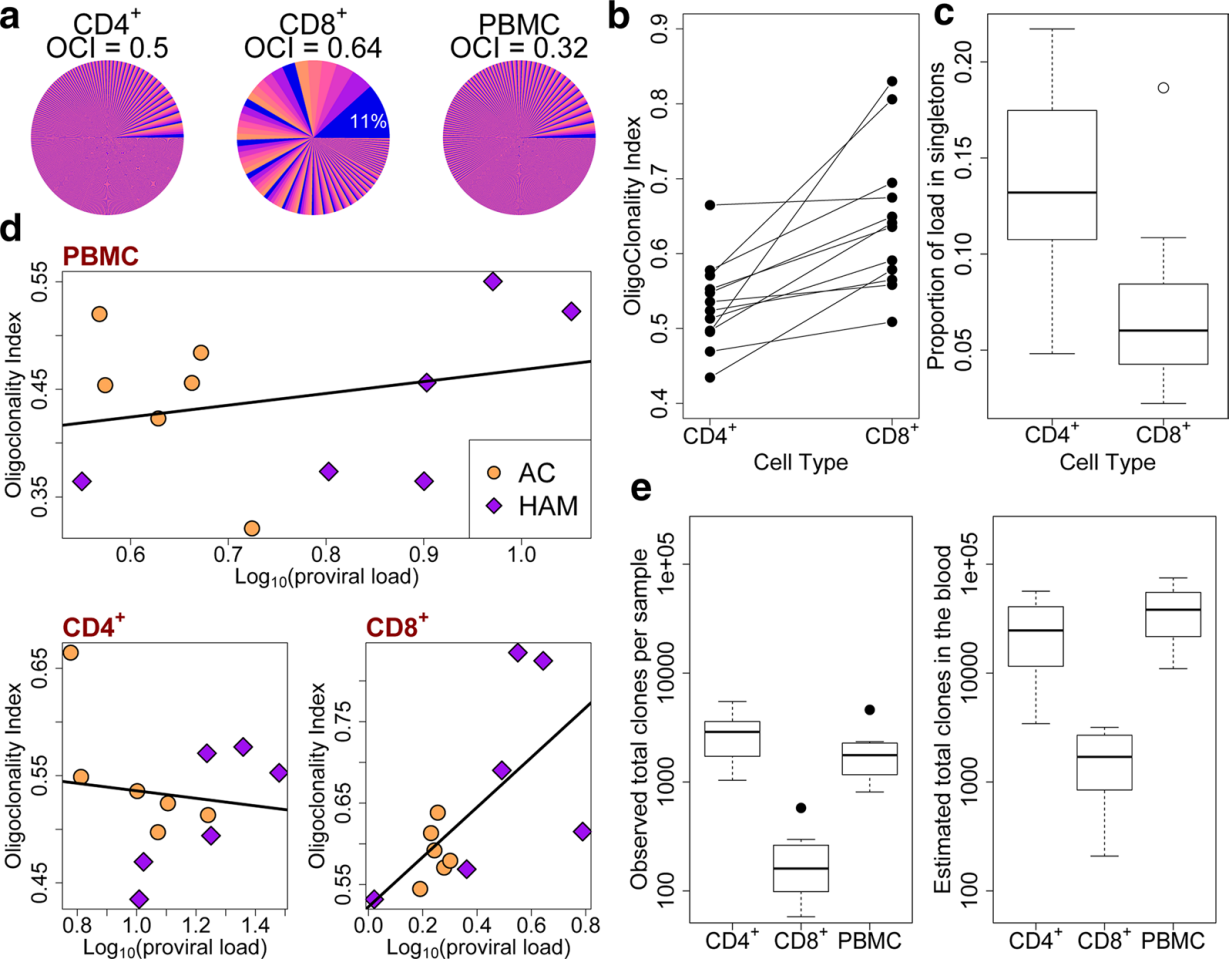
HTLV-1 clone frequency distribution in sorted CD4^+^ and CD8^+^ cells. **a** Representative example of the difference in clonal distribution between CD4^+^ (*left*), CD8^+^ (*middle*) and unsorted (*right*) cells. Each slice of the pie chart represents a single observed clone, and the width of the slice is proportional to its relative abundance in the respective subject. The most abundant CD8^+^ clone constituted over 10 % of the load in CD8^+^ cells in this subject. *OCI* oligoclonality index [15]. **b** The HTLV-1 oligoclonality index was significantly higher in CD8^+^ cell samples than in CD4^+^ cell samples (p = 0.0005, paired Wilcoxon signed rank test). **c** The proportion of the load present in singletons (clones detected only once per sample) was significantly higher in CD4^+^ cells than in CD8^+^ cells (p = 0.002, Mann–Whitney test). **d** The oligoclonality index in CD8^+^ cells was significantly correlated with the proviral load in CD8^+^ cells (p = 0.017, Spearman’s rank correlation). No such correlation was observed in CD4^+^ cells or in unsorted PBMCs. **e** The observed total clone number (*left panel*) and estimated total number of clones in the blood (*right panel*) for sorted CD4^+^, CD8^+^ cells and unsorted PBMCs

We examined the distribution of the proviral load among the clones in each respective cell population. The CD8^+^ cell population contained fewer infected cells (Additional file 4: Figure S3A) and fewer clones (Additional file 4: Figure S3B) than the CD4^+^ population. The observed difference in the clone frequency distribution between the CD4^+^ samples and the CD8^+^ samples is illustrated in Fig. 2a (see also Additional file 5: Figure S4).

The oligoclonality index [15] is a measure of non-uniformity in the clone frequency distribution. The median oligoclonality index of the CD8^+^ samples was 0.60 (range 0.53–0.83), significantly higher than that of the CD4^+^ samples (median 0.53, range 0.43–0.66) (p = 0.0005, Wilcoxon signed rank test, Fig. 2b). That is, there was a less uniform clone frequency distribution—a greater degree of oligoclonality—in the CD8^+^ clones than in CD4^+^ clones. Whereas the infected CD4^+^ cell population often consisted of one or few highly abundant clones and a large number of clones each of which was observed only once, the CD8^+^ population contained a significantly smaller total number of clones (p < 0.0001, Mann–Whitney); and a much smaller proportion of the load in CD8^+^ cells was made up by singletons (clones observed only once) than in the CD4^+^ population (Fig. 2c).

We found, as observed before [15], no correlation between oligoclonality index and proviral load either in the unsorted PBMCs or in the CD4^+^ cells. In the CD8^+^ cells, however, there was a significant positive correlation (p = 0.017, Spearman’s rank correlation) between the oligoclonality and proviral load in CD8^+^ cells (Fig. 2d).

The total number of clones observed in the CD4^+^ samples was significantly higher than in sorted CD8^+^ samples. However, it was also higher than the number in the unsorted PBMC samples. We hypothesize that this is due to enrichment of infected cells from small clones by cell sorting. We used the recently developed DivE method to estimate the total number of clones (clonal diversity) [24] in the blood. The estimated total number of CD8^+^ clones in the circulation was approximately tenfold less than that of CD4^+^ clones (Fig. 2e).

### HTLV-1-infected CD8^+^ clones are over-represented among the most abundant clones in non-malignant HTLV-1 infection

**Fig. 3:**
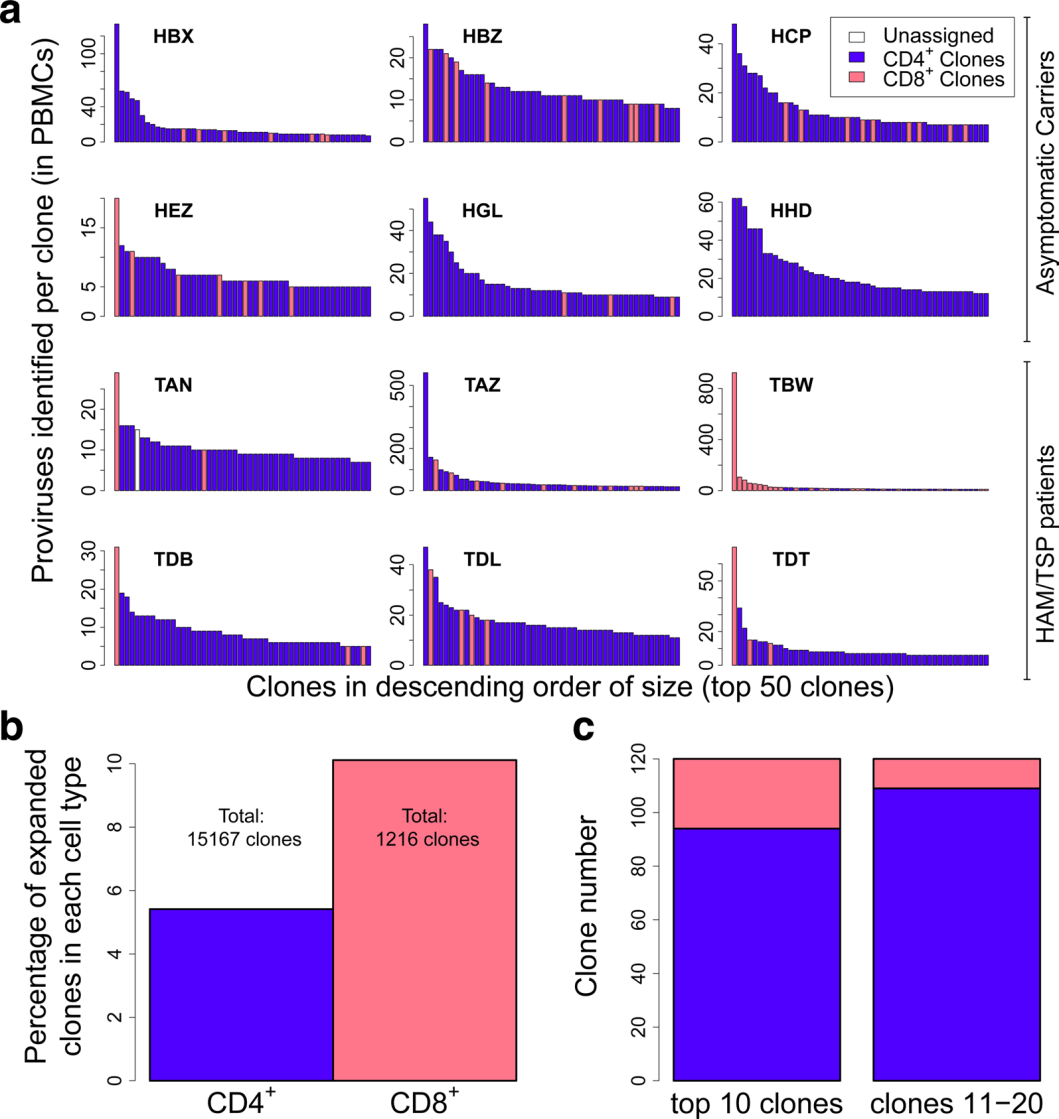
CD8^+^ clones are over-represented among the most abundant HTLV-1-infected clones in the blood. The clones detected by integration site analysis of PBMC samples from 12 HTLV-1-infected subjects were identified as either CD4^+^ or CD8^+^ if they were re-detected in the sorted samples. **a** The 50 most abundant clones are shown for each patient. For each clone, the number of proviruses detected is shown). The CD8^+^ clones (*pink*) included the most abundant single clone in 5/12 patients, and at least one of the three most abundant clones of 8/12 patients (including all HAM/TSP patients). **b** CD8^+^ clones are significantly more likely to be of high abundance, i.e. clones with greater than 1 cell per 10000 PBMCs (p < 0.0001, Fisher’s exact test). **c** Clones were ranked in descending order of abundance. CD8^+^ clones were significantly more likely to be present among the top 10 clones than among the next 10 (clones 11–20) (p = 0.012, Fisher’s exact test)

To quantify the relative contributions of CD4^+^ cells and CD8^+^ cells to the clone frequency distribution in unsorted PBMCs, we compared the clones identified in the CD4^+^ and CD8^+^ populations to the clones found in the unsorted PBMCs. The CD4^+^ or CD8^+^ phenotype of the 50 most abundant clones from each of the 12 subjects is shown in Fig. 3a. The phenotype of only one of the 600 clones was not initially identified in this way; we identified it to be a CD8^+^ clone by sequence similarity. Unexpectedly, in 5 out of the 12 infected subjects (including 4 of 6 HAM/TSP patients) the most abundant single clone was CD8^+^. In 8 of the 12 subjects (including all HAM/TSP patients) a CD8^+^ clone was present among the 3 most abundant clones. An extreme case was subject TBW (known to have a distorted CD4^+^/CD8^+^ ratio; see Table 1), in whom the proviral load was dominated by a large number of CD8^+^ clones, including the largest clone which represented over 15 % of the load.

To test whether CD8^+^ clones were more likely to be present among the most abundant clones than expected by chance, we compared the proportion of clones with an absolute abundance of more than 1 copy per 10,000 PBMCs between CD4^+^ and CD8^+^ clones (Fig. 3b). This proportion was significantly greater in CD8^+^ clones (~10 %) than in the CD4^+^ clones (~5 %; p < 0.0001, Fisher’s exact test). Ranking all clones in descending order of abundance (Fig. 3c), CD8^+^ clones were found more often among the 10 most abundant clones (clone 1–10) than in the next 10 (clones 11–20; p = 0.012, Fisher’s exact test).

### The abundant CD4^+^ and CD8^+^ HTLV-1-infected T-cell clones are long-lived in vivo

**Fig. 4:**
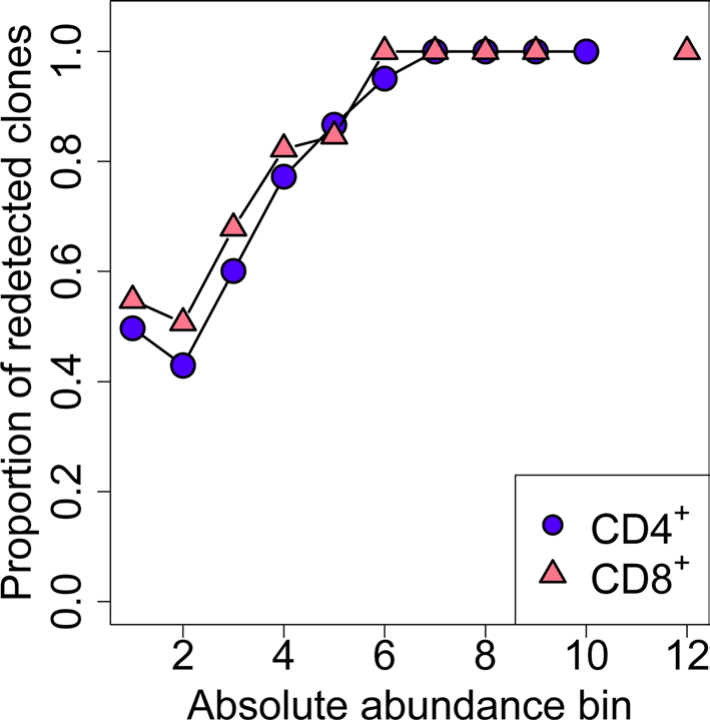
Expanded CD8 and CD4 clones are long-lived. HTLV-1 infected clones from 12 subjects were compared at two independent timepoints (the one studied above, and a second one at a median interval of 3.4 years). Clones here were grouped into bins of increasing log(absolute abundance). Only clones that were identified as CD4^+^ or CD8^+^ are shown here. For each abundance bin, the CD4^+^ and CD8^+^ clone data from each patient were compared to test the proportion of clones that were redetected at the second timepoint. Abundant clones (either CD4^+^ or CD8^+^) were likely to be redetected at a second timepoint. Within each abundance bin, CD8^+^ clones were redetected at least as frequently as CD4^+^ clones

In a previous study of the long-term survival of HTLV-1 clones in PBMCs we observed that a large proportion of the proviral load is made up of clones which can be redetected in the blood over many years of infection [15]. Here, we wished to test whether the highly abundant CD8^+^ clones are maintained over time in the blood or whether they are succeeded (replaced) by other clones. We analysed the integration sites in PBMC samples taken from each subject at a second timepoint, taken at a median interval of 3.4 years before or after the first sample analysed (range: 1.9–4.3 years). Sixty-four percent of CD4^+^ clones were redetected in samples taken at both timepoints, compared with 74 % of CD8^+^ clones. Because the abundance of a given clone determines its chance of redetection (Additional file 6: Figure S5A), we compared the proportion of redetected clones in bins of absolute clone abundance. As expected, the most abundant clones were consistently redetected at the second timepoint for both CD4^+^ and CD8^+^ clones; in the less abundant clones, CD8^+^ clones were redetected at least as frequently as CD4^+^ clones (Fig. 4, Additional file 6: Figure S5B).

### HTLV-1 infection alters the frequency of CD8^+^ cells but not CD4^+^ cells in peripheral blood

**Fig. 5:**
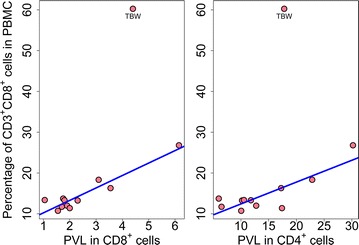
Correlation between proviral load in CD8^+^ cells and the proportion of CD8^+^ cells in PBMCs. The proviral load (PVL, copies per 100 cells) in CD8^+^ cells was strongly correlated with the proportion of CD8^+^ cells in PBMCs. The proportion of CD8^+^ cells in PBMCs is plotted against the proviral load in CD8^+^ cells (*left panel*; p = 0.02, Spearman’s rank correlation) and CD4^+^ cells (*right panel*; p = 0.06, Spearman’s rank correlation). The linear regression line was calculated excluding the CD4^+^ lymphopenic subject TBW (see text)

The proportion of the proviral load carried by CD8^+^ cells is determined by the load in the CD8^+^ cells and by the total frequency of CD8^+^ cells in the circulation. We found that the proviral load in CD8^+^ cells was positively correlated with the percentage of CD8^+^ T cells in PBMCs (Fig. 5, left panel) (p = 0.02, Spearman rank correlation). There was a positive trend of marginal significance between the proviral load in CD4^+^ cells and the percentage of CD8^+^ T cells in PBMCs (p = 0.06, Fig. 5, right panel). A similar positive trend of marginal significance was observed between the proviral load in CD8^+^ and CD4^+^ cells and the percentage of CD8^+^ T cells within the T-cell population (p = 0.06 and 0.07, respectively; Additional file 7: Figure S6A). There was no correlation between the proviral load in CD8^+^ or CD4^+^ cells and the percentage of CD4^+^ cells within PBMCs (Additional file 7: Figure S6B), suggesting that there is virus-driven selective expansion of CD8^+^ cells.

## Discussion

HTLV-1 primarily infects CD4^+^ T-cells in vivo, and the great majority of ATL cases are CD4^+^ [25]. The smaller, HTLV-1-infected CD8^+^ cell population has not been intensely studied. Small numbers of other cell types such as monocytes [26] may be infected. However, our data show that other cell types were unlikely to be present in the clonally expanded populations of HTLV-1-infected cells, because almost all (99.7 %) of the most highly abundant clones were identified as CD4^+^ or CD8^+^ cells. Small numbers of CD4^+^ monocytes might have been included in the sorted CD4^+^ fraction, but this number is likely to be very small because monocytes are not efficiently selected by magnetic bead sorting owing to their lower expression of CD4^+^ [27].

Our results show that HTLV-1-infected CD8^+^ cell clones make an unexpectedly large impact on the clone frequency distribution observed in the blood. While the CD8^+^ clones represent only a minority (median 5 %) of the proviral load, they are often highly represented among the most abundant clones in the blood, and among the most long-lived clones (across all clone abundances).

Comparison of the clone frequency distribution of proviruses between infected CD8^+^ and CD4^+^ cells revealed significant differences between the two populations. Whereas proviruses in infected CD4^+^ cells were present in a very large number of clones, each of which was often of low abundance, the clones observed in CD8^+^ T cells were often highly abundant. The oligoclonality index was significantly higher in CD8^+^ cell samples than in CD4^+^ cell samples and the proportion of the proviral load carried by low-abundance clones (clones observed only once) was significantly higher among CD4^+^ cell samples.

Previous investigations of HTLV-1 clonality showed no correlation between the HTLV-1 proviral load and the oligoclonality index in non-malignant HTLV-1 infection, but the correlation was significant in malignant HTLV-1 infection [15, 17, 20]. This observation was repeated here in the unsorted PBMCs; however, there was a significant positive correlation between the oligoclonality index in CD8^+^ cells and the proviral load in these cells. This observation suggests that the ratio of infectious spread (infection of new clones) to mitotic spread (proliferation of infected cells by increased mitotis or reduced apoptosis) differs between CD4^+^ and CD8^+^ cells during chronic HTLV-1 infection.

It is not known what mechanisms contribute to the difference in the degree of clonal expansion between the two cell types in HTLV-1 infection. We previously showed that the genomic integration site of the provirus plays a role in driving or silencing proviral gene expression, which in turn drives clonal expansion of CD4^+^ cells [15, 18]. However, infected CD8^+^ cells can express high levels of Tax and can then be killed by Tax-specific CD8^+^ cells [11, 28]. It is not known whether Tax-expressing CD4^+^ cells are killed as efficiently as Tax-expressing CD8^+^ cells. Proliferation of HTLV-1-infected CD8^+^ cells may be selectively enhanced by their response to specific antigen, such as those expressed by other persistent viruses or, perhaps most likely, antigens of HTLV-1 itself.

Oligoclonal expansion of Tax-specific CTLs has been reported [29], but it was not known whether these expanded clones are also infected by the virus. The notion that HTLV-1-infected, HTLV-1-specific CTLs grow to high abundance in the circulation is consistent with the previous observations that HTLV-1-specific CTLs are preferentially infected with HTLV-1 [11] and that the frequency of HTLV-1-specific CTLs is correlated with the proviral load [30, 31].

Sibon et al. [32] cultured clones of HTLV-1-infected and uninfected CD4^+^ and CD8^+^ cells, and concluded that the more vigorous expansion observed in infected CD8^+^ cells in vitro was due to a reduction in apoptosis rather than increased proliferation. If this is also the case in vivo, it could explain how CD8^+^ clones proliferate and persist, with a low risk of generating the somatic mutations that contribute to malignant transformation [33]. IL-15 has been shown to protect Tax-specific cells from apoptosis [34]. HTLV-1 Tax upregulates both IL-15 and its receptor [35, 36], and an increase in IL-15 mRNA has been observed in HAM/TSP patients [37]. Thus, HTLV-1 may exploit a normal IL-15-dependent pathway for the maintenance of memory CD8^+^ cells [38] to maintain its own infected CD8^+^ cell population. Cytokine-dependent proliferation of infected CD8^+^ cells could also explain the observation made in this study that the proportion of CD8^+^ T cells in the PBMCs was positively correlated with the viral burden in the CD8^+^ cells: abundant IL-15 secreted by infected cells could also drive proliferation of uninfected CD8^+^ T cells.

Previously [20] we reported the clone frequency distribution of HTLV-2, a virus closely related to HTLV-1; HTLV-2 is mainly found in CD8^+^ cells in infected individuals. Unlike HTLV-1, HTLV-2 does not cause leukemia or lymphoma. The findings here and those observed in HTLV-2 infection share several similarities. As we observed in HTLV-1-infected CD8^+^ cells, HTLV-2 infection was characterized by a high oligoclonality index due to a small number of abundant clones in the absence of malignancy. The oligoclonality index in HTLV-2 was significantly correlated with the proviral load, as observed here in HTLV-1-infected CD8^+^ (but not CD4^+^) cells. These observations suggest that physiological differences between CD8^+^ cells and CD4^+^ cells contribute to the observed differences in the clone frequency distribution between the two infected cell populations.

HTLV-1 infection is associated with a strong, constitutively active anti-HTLV-1 cytotoxic T-lymphocyte (CTL) response. Virtually all individuals with non-malignant HTLV-1-infection possess CTLs specific to Tax peptides [39, 40]. The most immunogenic peptide encoded by the virus is Tax_11-19_, which is efficiently presented in the context of HLA-A2 [41, 42]. Despite its high immunogenicity, this peptide is highly conserved. HTLV-2 encodes a nearly identical immunodominant peptide [43]. It is possible that this highly immunogenic peptide provides a selective advantage to the virus by driving proliferation of infected, antigen-specific T cells.

We conclude that the combined mitotic and antigenic effects of Tax in maintaining proliferation of infected CD8^+^ T cells may outweigh the negative selection by Tax-specific CTL-mediated killing of the infected CD8^+^ T cells.

We recently showed that the HTLV-1 proviral load, the strongest predictor of the risk of both inflammatory disease (HAM/TSP) and malignant disease (ATL), does not correlate with the degree of clonal expansion (the oligoclonality index), but rather with the total number of HTLV-1-infected clones. We also found, in an analysis of the integration site preferences among ~200 ATL patients, that the malignant ATL clones resemble the low-abundance clones more closely than the intermediate-abundance clones that have undergone oligoclonal proliferation [16]. ATL can arise by the rapid emergence (within 18 months) of a previously rare clone, outgrowing the pre-existing oligoclonally expanded clones [44]. Finally, HTLV-2 was found in highly abundant clones in vivo, but this virus does not cause leukemia or lymphoma [20]. However, malignant diseases of CD8^+^ T cells are generally rarer than those of CD4^+^ T cells [45], suggesting that the cell type determines the risk of malignancy. The viral mechanisms that cause clonal proliferation of HTLV-1-infected cells may be distinct from those that lead to malignant transformation.

## Conclusions

The observation that HTLV-1 causes oligoclonal proliferation of infected cells led to a widespread assumption that the oligoclonal proliferation predisposes to leukemogenesis. Here we show that contrary to that assumption, the cells that undergo the greatest clonal expansion in HTLV-1 infection are the HTLV-1-infected CD8^+^ cells. However, cases of CD8^+^ ATL are very rare. The results of the present study therefore add further to the conclusion [44] that oligoclonal T-cell proliferation per se does not predispose to malignant disease in HTLV-1 infection. Further work is needed to determine the mechanisms involved with the selective expansion of certain CD8^+^ clones, and their potential role in HTLV-1-associated disease.

## Methods

### Ethics statement

Blood samples and anonymized patient information were obtained through the Communicable Diseases Tissue Bank at Imperial College, approved by the UK National Research Ethics Service (NRES reference 09/H0606/106). Samples were donated by HTLV-1-infected subjects attending the National Centre for Human Retrovirology, St Mary’s Hospital, Imperial College Healthcare NHS Trust, London after giving written informed consent.

### Cells and samples

PBMC samples from 12 HTLV-1-infected individuals were analysed. See Table 1 for details of samples used. The 12 subjects included 6 patients with HAM/TSP and 6 asymptomatic carriers. ACs with a high PVL were selected; if the PVL is less than 0.1 % the number of proviruses sampled does not give adequate statistical power. One of the ACs (HGL) was diagnosed with HAM/TSP approximately 1 year after the sample was taken.

PBMCs were isolated from blood using Histopaque-1077 (Sigma-Aldrich) and cryopreserved in fetal bovine serum (Gibco) containing 10 % dimethylsulfoxide (Sigma-Aldrich). DNA was extracted from sorted or unsorted cells using the DNeasy Blood and Tissue Kit (Qiagen) according to the manufacturer’s protocol, and eluted in Qiagen EB buffer.

### Cell sorting

Uncultured, unfixed, cryopreserved PBMCs were sorted sequentially on the basis of CD4 and CD8 expression, using magnetic beads (Miltenyi). First, CD4^+^ cells were positively selected, and CD8^+^ cells were then selected from the CD4^−^ fraction. The cells were passed twice over sorting columns at each stage, to maximize purity and recovery.

Purity testing and analysis of cell population frequency in unsorted samples was carried out using flow cytometry, after fixation and staining for surface expression of CD3 (clone UCHT1, eBioscience), CD4 (clone RPA-T4, eBioscience) and CD8 (clone SFCI21Thy2D3, Beckman Coulter).

### Clonality analysis

Genomic DNA was extracted from both the sorted and the unsorted cell populations, and from unsorted PBMCs at a second time point (median time difference 3.4 years) where available. Proviral integration sites were mapped and quantified as previously described [15]. Sequencing of amplified integration sites was done using the Illumina GAII, HiSeq or MiSeq platforms, using 50-base paired-end reads and a 6-base barcode to allow multiplexing. Sequence data were aligned against a combined reference of the human genome (hg18) and the HTLV-1 genome sequence using an Eland implementation of CASAVA software (Illumina), then filtered and quantified for clone abundance using in-house software as previously described. See Additional file 2: Table S2 and Additional file 8 for details of the sequencing results.

Based on the assumption that HTLV-1 infects differentiated, mature CD4 or CD8 single-positive cells, we attributed any integration site found in both CD4^+^ and CD8^+^ cells to the cell subpopulation (CD4^+^ or CD8^+^) in which it was identified with a higher frequency (greater number of proviruses). The frequency of these putative contaminating clones was significantly inversely correlated with the measured purity of the sample, and significantly positively correlated with the frequency of the contaminating population and its share of the load (not shown).

### Statistical analysis

Data analysis was carried out using R (http://www.R-project.org/ [46]). The oligoclonality index was calculated using the package reldist [47]. Unless specified otherwise, non-parametric statistical tests were used. A result was considered statistically significant when p < 0.05 (two-tailed).

The total number of clones present in the circulation was estimated using the DivE estimator [24]. DivE fits multiple mathematical models to individual-based rarefaction curves; such curves plot the expected number of clones against the number of infected cells sampled. Numerical criteria are then used to score models on their ability to accurately estimate additional data. The best-performing models are extrapolated to estimate the total number of clones in the blood, based on the proviral load in the respective subject.

## Additional files

10.1186/s12977-015-0221-1 Shared clones in sorted, unsorted cells. For each panel, the numbers in the intersects of the Venn diagram represents the number of clones shared between the sorted CD4^+^ (left) or CD8^+^ (right) cells and the unsorted cells (middle).
10.1186/s12977-015-0221-1 Clonality analysis–additional data tables.
10.1186/s12977-015-0221-1 Estimation of CD8^+^ contribution to load is consistent using multiple methods. HTLV-1-infected CD4^+^ and CD8^+^ cells were separated by magnetic bead sorting and then analysed for their HTLV-1 proviral load and integration site frequency. The contribution of CD8^+^ cells to the proviral load was similar when estimated by two different methods: either from proviral load measurements or from the cumulative proportion of proviruses detected in CD8^+^ clones in the PBMC (p<0.0001, r=0.969, Pearson linear regression).
10.1186/s12977-015-0221-1 CD8^+^ samples are characterized by a small number of unique clones. Integration sites from sorted CD4^+^, CD8^+^ cells and unsorted PBMCs were identified. (A) The total number of proviruses identified in each sample (across all clones). (B) The total number of unique clones (unique integration sites) identified in each sample.
10.1186/s12977-015-0221-1 HTLV-1 clone frequency distribution in unsorted, and sorted CD4^+^ and CD8^+^ cells. The observed difference in clonal distribution across all patients between CD4^+^ (left), CD8^+^ (middle) and unsorted (right) cells. For each panel (for each subject), each slice of the pie chart represents a single observed clone, and the width of the slice is proportional to its relative abundance in the respective subject. The most abundant CD8^+^ clone constituted over 10% of the load in CD8^+^ cells in this subject. OCI - oligoclonality index.
10.1186/s12977-015-0221-1 Expanded CD4^+^ and CD8^+^ clones are more frequently redetected at a second timepoint. HTLV-1-infected clones from 12 subjects were compared at two independent timepoints (the one studied above, and a second at a median interval of 3.4 years). A) Clones that were re-detected had a significantly higher (p<0.0001, Mann-Whitney test) absolute abundance (proviral copies / 10000 PBMCs) than clones that were not redetected. B) For each abundance bin (increasing exponentially in absolute abundance), CD4^+^ and CD8^+^ T cells from each patient were compared to test the proportion of clones that were redetected at the second timepoint. Where there were sufficient points for comparison, no significant difference in the frequency of redetection was found between CD4^+^ and CD8^+^ clones (Wilcoxon signed rank test).
10.1186/s12977-015-0221-1 The proviral load in sorted T cells does not correlate with the proportion of CD8^+^ cells in T cells or the proportion of CD4^+^ cells in PBMCs. No significant correlation was found between the proviral load in CD8^+^ cells (left) or the proviral load in CD4^+^ cells (right) and (A) the percentage of CD8^+^ cells in the CD3^+^ population (p=0.06 and p=0.07, respectively, Spearman’s rank correlation) or (B) the proportion of CD4^+^ cells in PBMCs (p=0.89 and p=0.97, respectively, Spearman’s rank correlation). The linear regression line was calculated excluding the CD4^+^ lymphopenic subject TBW (see text). PVL – proviral load (copies per 100 cells).
10.1186/s12977-015-0221-1 Identified integration sites.

## References

[1] Gessain A, Cassar O (2012). Epidemiological Aspects and World Distribution of HTLV-1 Infection. Front Microbiol.

[2] Matsuzaki T, Nakagawa M, Nagai M, Usuku K, Higuchi I, Arimura K (2001). HTLV-I proviral load correlates with progression of motor disability in HAM/TSP: analysis of 239 HAM/TSP patients including 64 patients followed up for 10 years. J Neurovirol.

[3] Iwanaga M, Watanabe T, Utsunomiya A, Okayama A, Uchimaru K, Koh KR (2010). Human T-cell leukemia virus type I (HTLV-1) proviral load and disease progression in asymptomatic HTLV-1 carriers: a nationwide prospective study in Japan. Blood.

[4] Richardson JH, Edwards AJ, Cruickshank JK, Rudge P, Dalgleish AG (1990). In vivo cellular tropism of human T-cell leukemia virus type 1. J Virol.

[5] Nagai M, Brennan MB, Sakai JA, Mora CA, Jacobson S (2001). CD8(+) T cells are an in vivo reservoir for human T-cell lymphotropic virus type I. Blood.

[6] Jones KS, Fugo K, Petrow-Sadowski C, Huang Y, Bertolette DC, Lisinski I (2006). Human T-cell leukemia virus type 1 (HTLV-1) and HTLV-2 use different receptor complexes to enter T cells. J Virol.

[7] Kannian P, Yin H, Doueiri R, Lairmore MD, Fernandez S, Green PL (2012). Distinct transformation tropism exhibited by human T lymphotropic virus type 1 (HTLV-1) and HTLV-2 is the result of postinfection T cell clonal expansion. J Virol.

[8] Hattori T, Uchiyama T, Toibana T, Takatsuki K, Uchino H (1981). Surface phenotype of Japanese adult T-cell leukemia cells characterized by monoclonal antibodies. Blood.

[9] Tsukasaki K, Hermine O, Bazarbachi A, Ratner L, Ramos JC, Harrington W (2009). Definition, prognostic factors, treatment, and response criteria of adult T-cell leukemia-lymphoma: a proposal from an international consensus meeting. J Clin Oncol.

[10] Mahieux R, Gessain A (2007). Adult T-cell leukemia/lymphoma and HTLV-1. Curr Hematol Malig Reports.

[11] Hanon E, Stinchcombe JC, Saito M, Asquith BE, Taylor GP, Tanaka Y (2000). Fratricide among CD8(+) T lymphocytes naturally infected with human T cell lymphotropic virus type I. Immunity.

[12] Popovic M, Flomenberg N, Volkman DJ, Mann D, Fauci AS, Dupont B (1984). Alteration of T-cell functions by infection with HTLV-I or HTLV-II. Science.

[13] Mitsuya H, Jarrett RF, Cossman J, Cohen OJ, Kao CS, Guo HG (1986). Infection of human T lymphotropic virus-I-specific immune T cell clones by human T lymphotropic virus-I. J Clin Invest..

[14] Faller DV, Crimmins MA, Mentzer SJ (1988). Human T-cell leukemia virus type I infection of CD4^+^ or CD8^+^ cytotoxic T-cell clones results in immortalization with retention of antigen specificity. J Virol.

[15] Gillet NA, Malani N, Melamed A, Gormley N, Carter R, Bentley D (2011). The host genomic environment of the provirus determines the abundance of HTLV-1-infected T-cell clones. Blood.

[16] Cook LB, Melamed A, Niederer H, Valganon M, Laydon D, Foroni L (2014). The role of HTLV-1 clonality, proviral structure, and genomic integration site in adult T-cell leukemia/lymphoma. Blood.

[17] Gillet NA, Cook L, Laydon DJ, Hlela C, Verdonck K, Alvarez C (2013). Strongyloidiasis and infective dermatitis alter human T lymphotropic virus-1 clonality in vivo. PLoS Pathog.

[18] Melamed A, Laydon DJ, Gillet NA, Tanaka Y, Taylor GP, Bangham CR (2013). Genome-wide determinants of proviral targeting, clonal abundance and expression in natural HTLV-1 infection. PLoS Pathog.

[19] Niederer HA, Laydon DJ, Melamed A, Elemans M, Asquith B, Matsuoka M (2014). HTLV-1 proviral integration sites differ between asymptomatic carriers and patients with HAM/TSP. Virol J..

[20] Melamed A, Witkover AD, Laydon DJ, Brown R, Ladell K, Miners K (2014). Clonality of HTLV-2 in natural infection. PLoS Pathog.

[21] Gillet NA, Gutierrez G, Rodriguez SM, de Brogniez A, Renotte N, Alvarez I (2013). Massive depletion of bovine leukemia virus proviral clones located in genomic transcriptionally active sites during primary infection. PLoS Pathog.

[22] Maldarelli F, Wu X, Su L, Simonetti FR, Shao W, Hill S (2014). HIV latency. Specific HIV integration sites are linked to clonal expansion and persistence of infected cells. Science.

[23] Cook LB, Rowan AG, Melamed A, Taylor GP, Bangham CR (2012). HTLV-1-infected T cells contain a single integrated provirus in natural infection. Blood.

[24] Laydon DJ, Melamed A, Sim A, Gillet NA, Sim K, Darko S (2014). Quantification of HTLV-1 clonality and TCR diversity. PLoS Comput Biol.

[25] Kamihira S, Sohda H, Atogami S, Toriya K, Yamada Y, Tsukazaki K (1992). Phenotypic diversity and prognosis of adult T-cell leukemia. Leuk Res.

[26] Koyanagi Y, Itoyama Y, Nakamura N, Takamatsu K, Kira J, Iwamasa T (1993). In vivo infection of human T-cell leukemia virus type I in non-T cells. Virology.

[27] Filion LG, Izaguirre CA, Garber GE, Huebsh L, Aye MT (1990). Detection of surface and cytoplasmic CD4 on blood monocytes from normal and HIV-1 infected individuals. J Immunol Methods.

[28] Hanon E, Hall S, Taylor GP, Saito M, Davis R, Tanaka Y (2000). Abundant tax protein expression in CD4^+^ T cells infected with human T-cell lymphotropic virus type I (HTLV-I) is prevented by cytotoxic T lymphocytes. Blood.

[29] Utz U, Banks D, Jacobson S, Biddison WE (1996). Analysis of the T-cell receptor repertoire of human T-cell leukemia virus type 1 (HTLV-1) Tax-specific CD8^+^ cytotoxic T lymphocytes from patients with HTLV-1-associated disease: evidence for oligoclonal expansion. J Virol.

[30] Kubota R, Kawanishi T, Matsubara H, Manns A, Jacobson S (2000). HTLV-I specific IFN-gamma + CD8^+^ lymphocytes correlate with the proviral load in peripheral blood of infected individuals. J Neuroimmunol.

[31] Wodarz D, Hall SE, Usuku K, Osame M, Ogg GS, McMichael AJ (2001). Cytotoxic T-cell abundance and virus load in human immunodeficiency virus type 1 and human T-cell leukaemia virus type 1. Proc Biol Sci/The Royal Soc.

[32] Sibon D, Gabet AS, Zandecki M, Pinatel C, Thete J, Delfau-Larue MH (2006). HTLV-1 propels untransformed CD4 lymphocytes into the cell cycle while protecting CD8 cells from death. J Clin Invest..

[33] Tomasetti C, Vogelstein B (2015). Cancer etiology Variation in cancer risk among tissues can be explained by the number of stem cell divisions. Science.

[34] Azimi N, Nagai M, Jacobson S, Waldmann TA (2001). IL-15 plays a major role in the persistence of Tax-specific CD8 cells in HAM/TSP patients. Proc Natl Acad Sci USA.

[35] Azimi N, Brown K, Bamford RN, Tagaya Y, Siebenlist U, Waldmann TA (1998). Human T cell lymphotropic virus type I Tax protein trans-activates interleukin 15 gene transcription through an NF-kappaB site. Proc Natl Acad Sci USA.

[36] Mariner JM, Lantz V, Waldmann TA, Azimi N (2001). Human T cell lymphotropic virus type I Tax activates IL-15R alpha gene expression through an NF-kappa B site. J Immunol.

[37] Azimi N, Jacobson S, Leist T, Waldmann TA (1999). Involvement of IL-15 in the pathogenesis of human T lymphotropic virus type I-associated myelopathy/tropical spastic paraparesis: implications for therapy with a monoclonal antibody directed to the IL-2/15R beta receptor. J Immunol.

[38] Ku CC, Murakami M, Sakamoto A, Kappler J, Marrack P (2000). Control of homeostasis of CD8^+^ memory T cells by opposing cytokines. Science.

[39] Goon PK, Biancardi A, Fast N, Igakura T, Hanon E, Mosley AJ (2004). Human T cell lymphotropic virus (HTLV) type-1-specific CD8^+^ T cells: frequency and immunodominance hierarchy. J Infect Dis.

[40] Hilburn S, Rowan A, Demontis MA, MacNamara A, Asquith B, Bangham CR (2011). In vivo expression of human T-lymphotropic virus type 1 basic leucine-zipper protein generates specific CD8^+^ and CD4^+^ T-lymphocyte responses that correlate with clinical outcome. J Infect Dis.

[41] Koenig S, Woods RM, Brewah YA, Newell AJ, Jones GM, Boone E (1993). Characterization of MHC class I restricted cytotoxic T cell responses to tax in HTLV-1 infected patients with neurologic disease. J Immunol.

[42] Elovaara I, Koenig S, Brewah AY, Woods RM, Lehky T, Jacobson S (1993). High human T cell lymphotropic virus type 1 (HTLV-1)-specific precursor cytotoxic T lymphocyte frequencies in patients with HTLV-1-associated neurological disease. J Exp Med.

[43] Oliveira AL, Hayakawa H, Schor D, Leite AC, Espindola OM, Waters A (2009). High frequencies of functionally competent circulating Tax-specific CD8^+^ T cells in human T lymphotropic virus type 2 infection. J Immunol.

[44] Bangham CR, Cook LB, Melamed A (2014). HTLV-1 clonality in adult T-cell leukaemia and non-malignant HTLV-1 infection. Semin Cancer Biol.

[45] Swerdlow S, Campo E, Harris NL, Jaffe ES, Pileri S, Stein H et al. WHO classification of tumours of haematopoietic and lymphoid tissues. 4th edn. Lyon: Lyon: International Agency for Research on Cancer; 2008.

[46] R Development Core Team. R: A Language and Environment for Statistical Computing. Vienna, Austria: R Foundation for Statistical Computing; 2011.

[47] Handcock MS, Morris M (1999). Relative distribution methods in the social sciences. Statistics for social science and public policy.

