# Supplementary figures and images for: HTLV-1 drives vigorous clonal expansion of infected CD8+ T cells in natural infection
Source: Retrovirology. 2015 Nov 9;12:91. doi: 10.1186/s12977-015-0221-1 (PMC4640420; doi:10.1186/s12977-015-0221-1)

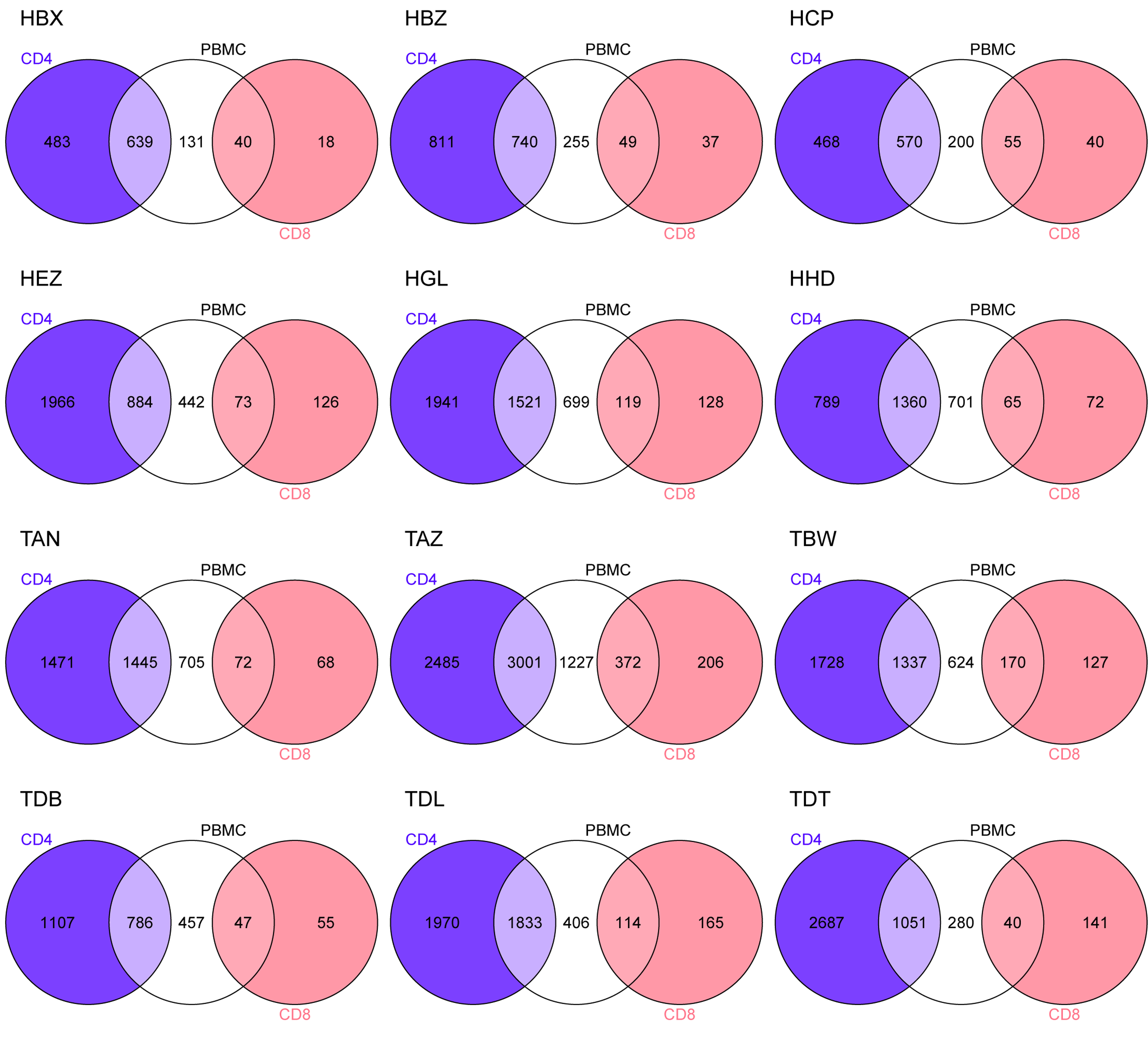

Supplement: Supplementary file 1 — 10.1186/s12977-015-0221-1 Shared clones in sorted, unsorted cells. For each panel, the numbers in the intersects of the Venn diagram represents the number of clones shared between the sorted CD4+ (left) or CD8+ (right) cells and the unsorted cells (middle). [file 12977_2015_221_MOESM1_ESM.tif]

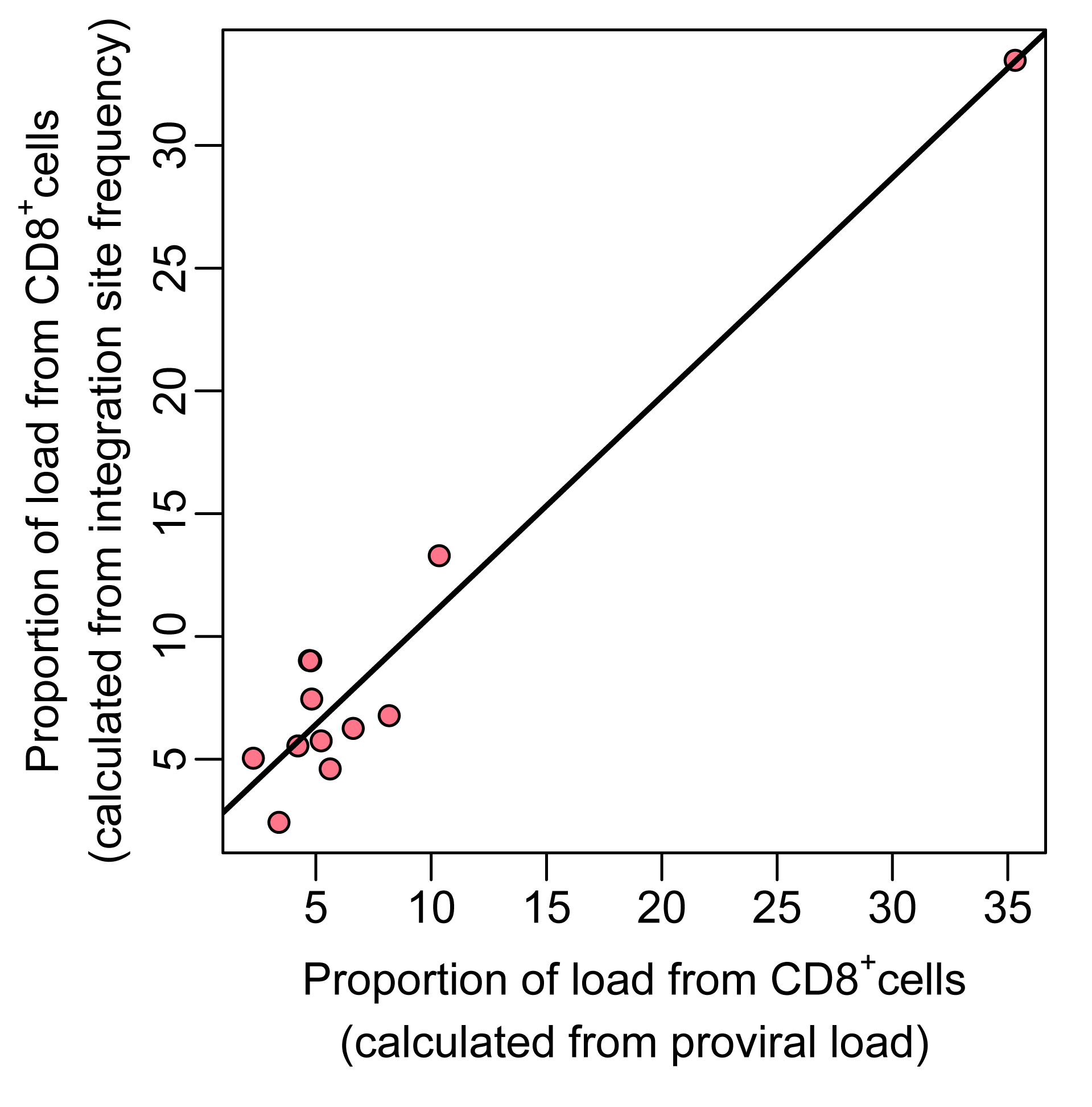

Supplement: Supplementary file 3 — 10.1186/s12977-015-0221-1 Estimation of CD8+ contribution to load is consistent using multiple methods. HTLV-1-infected CD4+ and CD8+ cells were separated by magnetic bead sorting and then analysed for their HTLV-1 proviral load and integration site frequency. The contribution of CD8+ cells to the proviral load was similar when estimated by two different methods: either from proviral load measurements or from the cumulative proportion of proviruses detected in CD8+ clones in the PBMC (p<0.0001, r=0.969, Pearson linear regression). [file 12977_2015_221_MOESM3_ESM.tif]

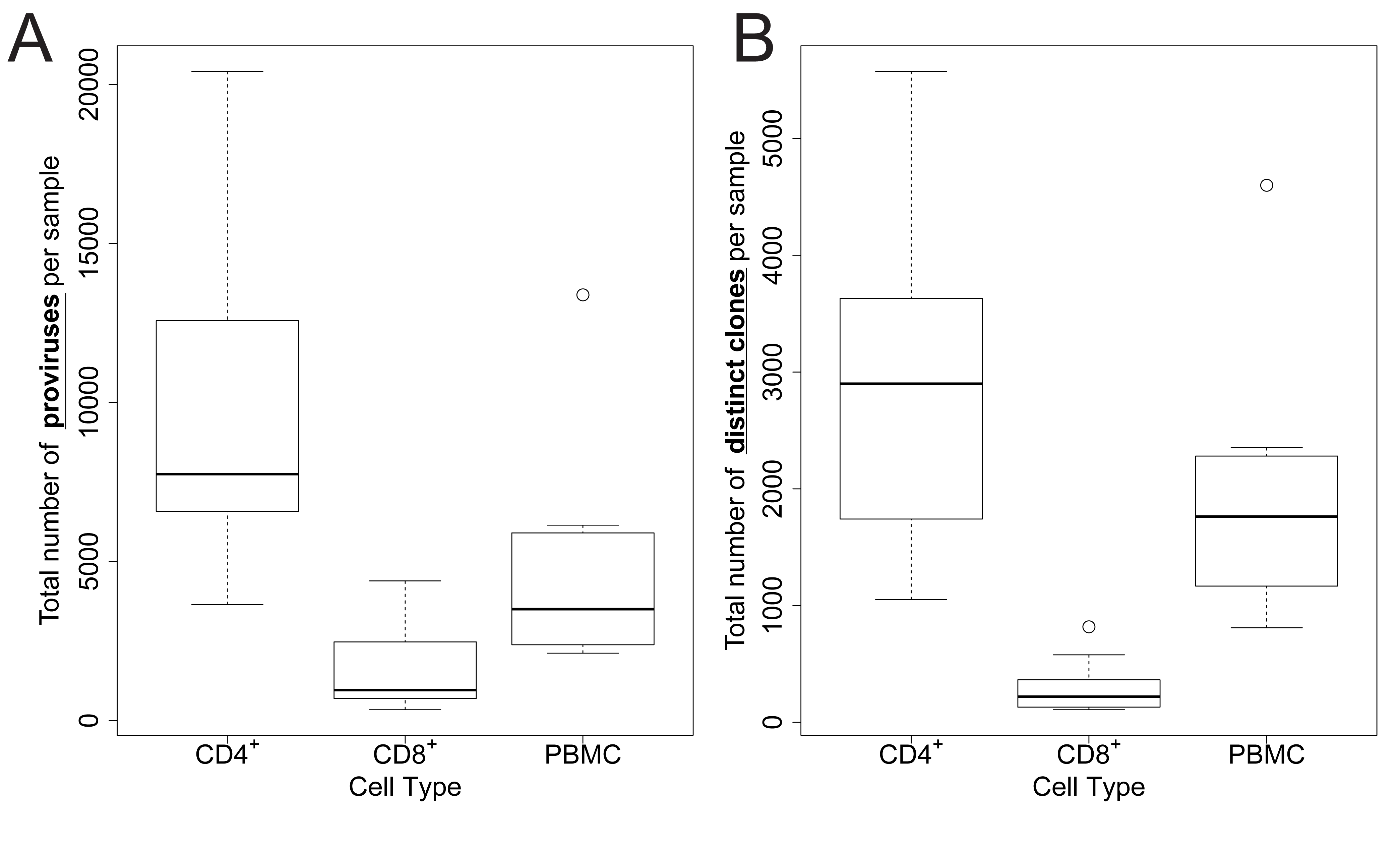

Supplement: Supplementary file 4 — 10.1186/s12977-015-0221-1 CD8+ samples are characterized by a small number of unique clones. Integration sites from sorted CD4+, CD8+ cells and unsorted PBMCs were identified. (A) The total number of proviruses identified in each sample (across all clones). (B) The total number of unique clones (unique integration sites) identified in each sample. [file 12977_2015_221_MOESM4_ESM.tif]

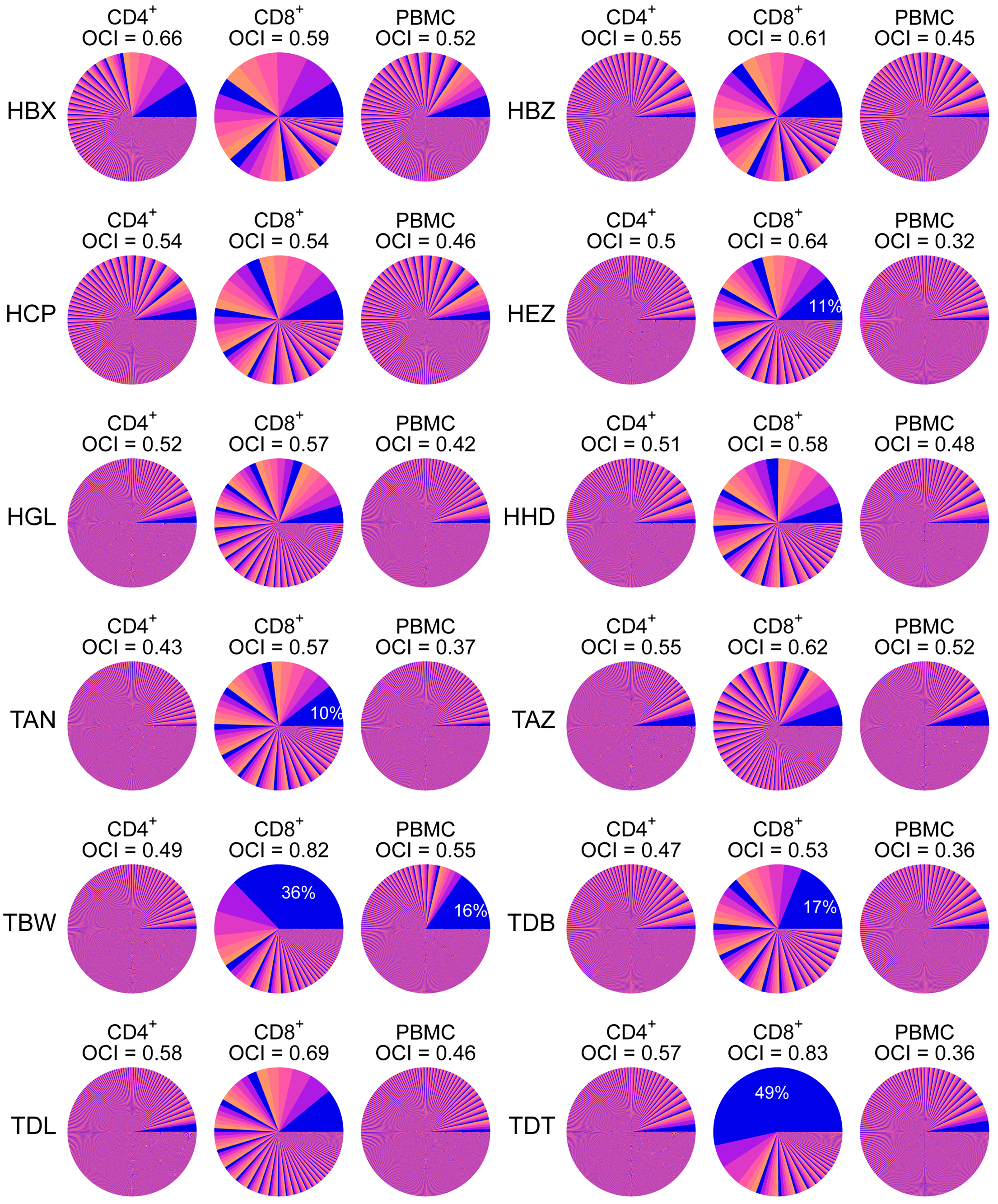

Supplement: Supplementary file 5 — 10.1186/s12977-015-0221-1 HTLV-1 clone frequency distribution in unsorted, and sorted CD4+ and CD8+ cells. The observed difference in clonal distribution across all patients between CD4+ (left), CD8+ (middle) and unsorted (right) cells. For each panel (for each subject), each slice of the pie chart represents a single observed clone, and the width of the slice is proportional to its relative abundance in the respective subject. The most abundant CD8+ clone constituted over 10% of the load in CD8+ cells in this subject. OCI - oligoclonality index. [file 12977_2015_221_MOESM5_ESM.tif]

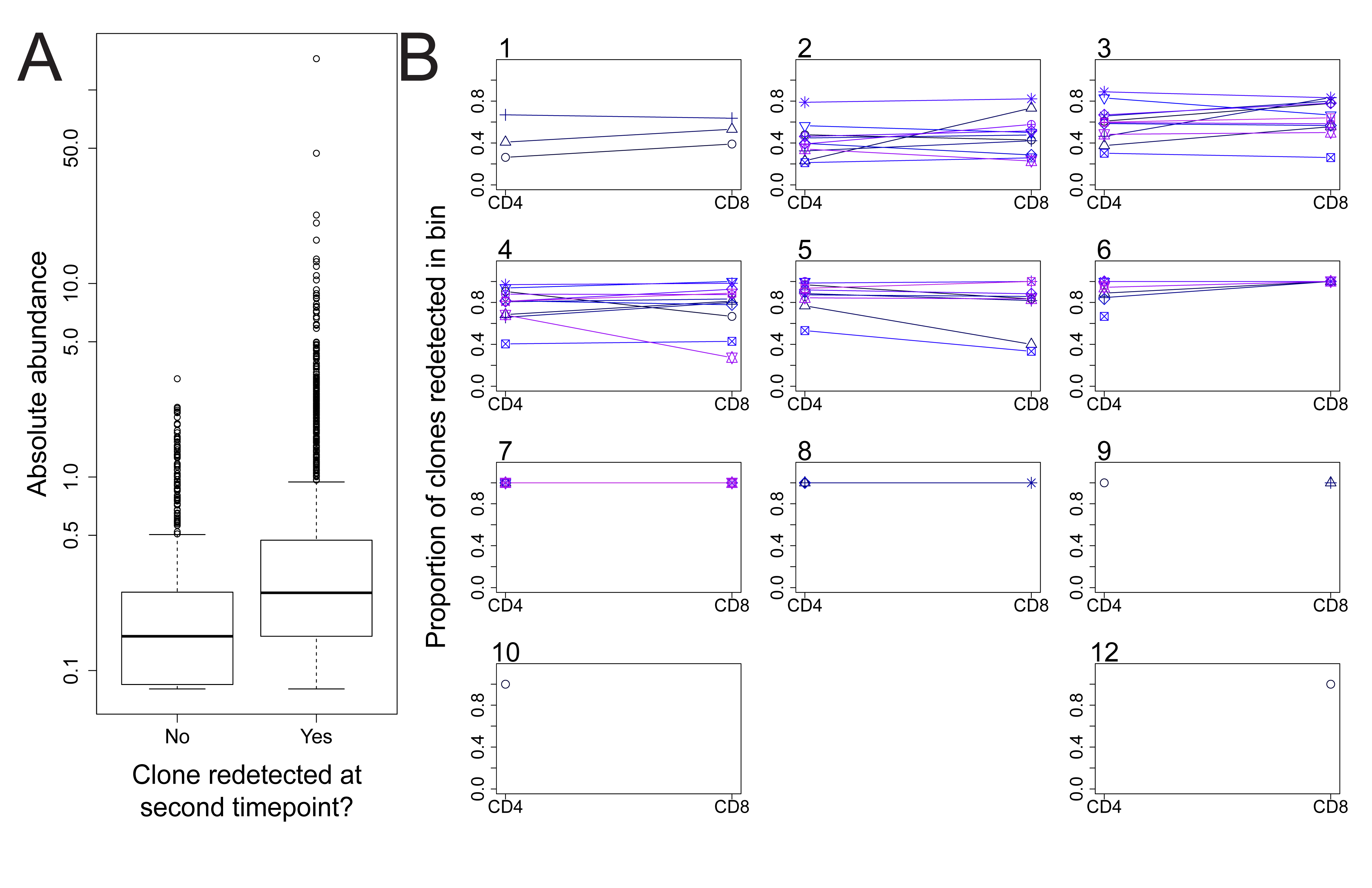

Supplement: Supplementary file 6 — 10.1186/s12977-015-0221-1 Expanded CD4+ and CD8+ clones are more frequently redetected at a second timepoint. HTLV-1-infected clones from 12 subjects were compared at two independent timepoints (the one studied above, and a second at a median interval of 3.4 years). A) Clones that were re-detected had a significantly higher (p<0.0001, Mann-Whitney test) absolute abundance (proviral copies / 10000 PBMCs) than clones that were not redetected. B) For each abundance bin (increasing exponentially in absolute abundance), CD4+ and CD8+ T cells from each patient were compared to test the proportion of clones that were redetected at the second timepoint. Where there were sufficient points for comparison, no significant difference in the frequency of redetection was found between CD4+ and CD8+ clones (Wilcoxon signed rank test). [file 12977_2015_221_MOESM6_ESM.tif]

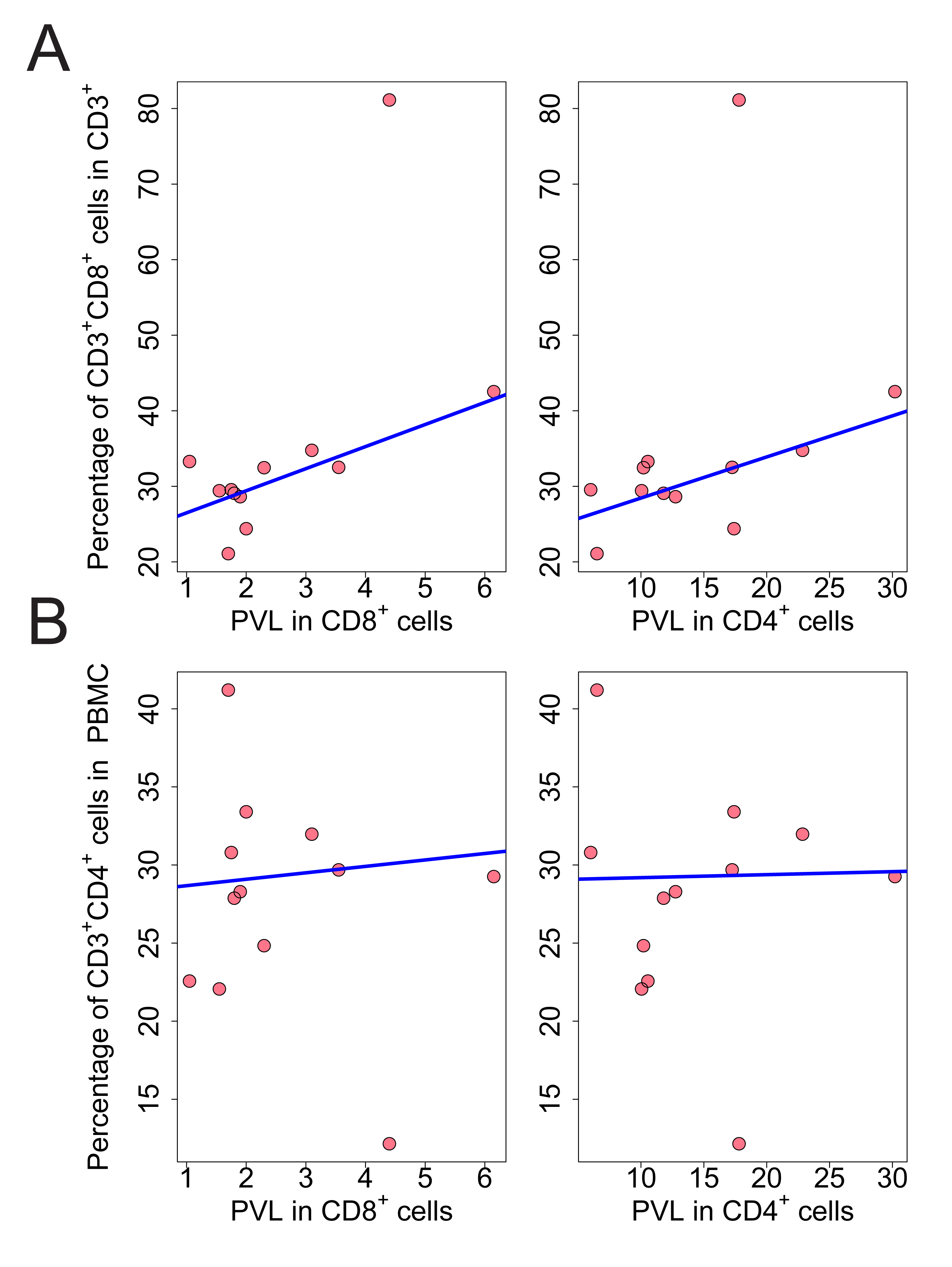

Supplement: Supplementary file 7 — 10.1186/s12977-015-0221-1 The proviral load in sorted T cells does not correlate with the proportion of CD8+ cells in T cells or the proportion of CD4+ cells in PBMCs. No significant correlation was found between the proviral load in CD8+ cells (left) or the proviral load in CD4+ cells (right) and (A) the percentage of CD8+ cells in the CD3+ population (p=0.06 and p=0.07, respectively, Spearman’s rank correlation) or (B) the proportion of CD4+ cells in PBMCs (p=0.89 and p=0.97, respectively, Spearman’s rank correlation). The linear regression line was calculated excluding the CD4+ lymphopenic subject TBW (see text). PVL – proviral load (copies per 100 cells). [file 12977_2015_221_MOESM7_ESM.tif]
