# Supplementary material for: HTLV-1 drives vigorous clonal expansion of infected CD8+ T cells in natural infection
Source: Retrovirology. 2015 Nov 9;12:91. doi: 10.1186/s12977-015-0221-1 (PMC4640420; doi:10.1186/s12977-015-0221-1)
Supplement: Supplementary file 2 — 10.1186/s12977-015-0221-1 Clonality analysis–additional data tables. [file 12977_2015_221_MOESM2_ESM.pdf]

Table S1 – the proviral burden in each cell population.

Subject code	Proviral Load (copies per 100 cells)			Proportion of load present in each population (%)	
	PVL[$CD4^+$]	PVL[$CD8^+$]	PVL[PBMC]	$CD4^+$	$CD8^+$
HBX	6.0	1.8	3.7	91.8	8.2
HBZ	6.5	1.7	3.8	95.2	4.8
HCP	10.1	1.6	4.6	95.3	4.7
HEZ	11.8	1.8	5.3	95.2	4.8
HGL	12.8	1.9	4.3	95.8	4.2
HHD	17.4	2.0	4.7	96.6	3.4
TAN	10.2	2.3	6.4	94.4	5.6
TAZ	30.2	6.2	11.3	89.7	10.4
TBW	17.8	4.4	9.4	64.7	35.3
TDB	10.6	1.1	3.6	97.7	2.3
TDL	22.9	3.1	8.0	94.8	5.2
TDT	17.3	3.6	8.0	93.4	6.6
**Median**	**12.3**	**2.0**	**5.0**	**95.0**	**5.0**

Table S2 – high-throughput sequencing results

Subject code	PBMC Reads	PBMC Clones	PBMC DNA input	CD8 Reads	CD8 Clones	CD8 DNA input	CD4 Reads	CD4 Clones	CD4 DNA input	2ND timepoint Reads	2ND timepoint Clones
HBX	138193	810	10	144776	58	6.76	415908	1122	10	1246701	744
HBZ	314815	1044	6.27	234507	86	3.4	164874	1551	10	1372006	576
HCP	545381	825	8.51	190367	95	3.12	357859	1038	7.34	923623	573
HEZ	553313	1399	4.23	457799	199	4.41	322207	2850	8.28	1016625	1581
HGL	206356	2339	10	299450	247	6.23	356660	3462	10	984807	1494
HHD	966688	2126	9.99	386828	137	6.76	435207	2149	9.99	839915	1542
TAN	442147	2222	9.99	351461	140	4.52	309701	2916	10	307611	954
TAZ	350247	4600	10	136354	578	9.99	217878	5486	10.02	986565	6149
TBW	232777	2131	10	595329	297	10.01	418217	3065	10	1696110	2157
TDB	156297	1290	6.57	257789	102	4.91	132461	1893	10	1075769	1256
TDL	265116	2353	10	264590	279	10	628060	3803	10.01	282235	1673
TDT	359200	1371	3.79	272218	181	5.3	592379	3738	10	1102441	2558

Reads – total number of sequencing reads (across all clones) for each sample. Clones – Total number of unique integration sites for each subject. DNA input – in mcg.

Table S3 – statistical analysis including/excluding outlier subject TBW

X-Y test

Test	Spearman including TBW	Spearman excluding TBW	Pearson including TBW	Pearson excluding TBW
PVL in CD8 vs contribution of CD8 to the load	0.01454	0.04782	0.0645	0.00407
PVL in PBMC vs contribution of CD8 to the load	0.02398	0.07594	0.06537	0.02038
PVL in PBMC vs PVL in CD8	< 2.2e-16	0.0004284	4.94e-07	3.995e-06
PVL in PBMC vs PVL in CD4	0.003617	0.01338	0.0002944	0.0002485
OCI vs PVL in PBMC	0.24626	0.59522	0.41834	0.81158
OCI vs PVL in CD4 cells	0.90374	0.79659	0.70043	0.80076
OCI vs PVL in CD8 cells	0.017305	0.040386	0.021142	0.073613
PVL in CD4 vs CD3+CD8+/PBMC	0.05903	0.1543	0.1885	0.0009671
PVL in CD8 vs CD3+CD8+/PBMC	0.02216	0.0706	0.02493	1.71e-05
PVL in CD4 vs CD3+CD4+/PBMC	0.9737	0.5952	0.7911	0.9392
PVL in CD8 vs CD3+CD4+/PBMC	0.8863	0.356	0.542	0.7503
PVL in CD4 vs CD3+CD8+/ CD3+	0.07068	0.1825	0.2201	0.01432
PVL in CD8 vs CD3+CD8+/ CD3+	0.05903	0.1728	0.04015	0.008898

Paired two groups

Test	Wilcoxon signed rank including TBW	Wilcoxon signed rank excluding TBW	t.test including TBW	t.test excluding TBW
OCI in CD8 cells vs OCI in CD4 cells	0.0004883	0.0009766	0.001644	0.0009364
Prop. singletons in CD8 cells vs Prop. singletons in CD4 cells	0.0004883	0.0009766	5.238e-05	4.086e-05
Number of observed clones (unique UIS) in CD8 cells vs CD4 cells	0.0004883	0.0009766	1.052e-05	4.18e-05
Estimated number of clones in the blood in CD8 cells vs CD4 cells	0.0004883	0.0009766	0.0003211	0.0008633

Contingency tables

Test	Fisher's exact test including TBW	Fisher's exact test excluding TBW	Chi-square including TBW	Chi-square excluding TBW
Expanded/non expanded clones in CD4/CD8	5.355e-10	0.0001565	1.996e-11	9.334e-05
Top 10/11-20 in CD4/CD8	0.01157	0.1287	0.01233	0.1301
